# Hydroethanolic Extract of *Polygonum aviculare* L. Mediates the Anti-Inflammatory Activity in RAW 264.7 Murine Macrophages Through Induction of Heme Oxygenase-1 and Inhibition of Inducible Nitric Oxide Synthase

**DOI:** 10.3390/plants13233314

**Published:** 2024-11-26

**Authors:** Chan Ho Jang, You Chul Chung, Ami Lee, Youn-Hwan Hwang

**Affiliations:** 1 Herbal Medicine Research Division, Korea Institution of Oriental Medicine (KIOM), Daejeon 34054, Republic of Korea; jyc8385@kiom.re.kr (Y.C.C.); dmb01367@kiom.re.kr (A.L.); 2 Korean Convergence Medical Science Major, KIOM School, University of Science & Technology (UST), Daejeon 34054, Republic of Korea

**Keywords:** *Polygonum aviculare* L., anti-inflammatory activity, antioxidative activity, HO-1, iNOS, quercetin, kaempferol

## Abstract

*Polygonum aviculare* L. (PAL), commonly known as knotgrass, has been utilized as a traditional folk medicine across Asian, African, Latin American and Middle Eastern countries to treat various inflammatory diseases, including arthritis and airway inflammation. Numerous medicinal herbs exert anti-inflammatory and antioxidative effects that are mediated through the activation of nuclear factor-erythroid 2-related factor 2 (Nrf2) and the inhibition of nuclear factor kappa B (NF-κB). However, the underlying molecular mechanisms linking the antioxidative and anti-inflammatory effects remain poorly understood. Heme oxygenase-1 (HO-1) is an antioxidant enzyme that catalyzes heme degradation, ultimately leading to the production of carbon monoxide (CO). Elevated levels of CO have been correlated with the decreased level of inducible nitric oxide synthase (iNOS). In this study, we examined whether HO-1 plays a key role in the relationship between the antioxidative and anti-inflammatory properties of PAL. The anti-inflammatory and antioxidative activities of PAL in an in vitro system were evaluated by determining NF-κB activity, antioxidant response element (ARE) activity, pro-inflammatory cytokine and protein levels, as well as antioxidant protein levels. To examine whether HO-1 inhibition interfered with the anti-inflammatory effect of PAL, we measured nitrite, reactive oxygen species, iNOS, and HO-1 levels in RAW 264.7 murine macrophages pre-treated with Tin protoporphyrin (SnPP, an HO-1 inhibitor). Our results demonstrated that PAL increased ARE activity and the Nrf2-regulated HO-1 level, exerting antioxidative activities in RAW 264.7 macrophages. Additionally, PAL reduced cyclooxygenase-2 (COX-2) and iNOS protein levels by inactivating NF-κB in lipopolysaccharide (LPS)-activated RAW 264.7 macrophages. Further investigation using the HO-1 inhibitor revealed that HO-1 inhibition promoted iNOS expression, subsequently elevating nitric oxide (NO) generation in LPS-activated RAW 264.7 macrophages treated with PAL compared to those in the macrophages without the HO-1 inhibitor. Overall, our findings suggest that HO-1 induction by PAL may exert anti-inflammatory effects through the reduction of the iNOS protein level. Hence, this study paves the way for further investigation to understand molecular mechanisms underlying the antioxidative and anti-inflammatory activities of medicinal herbs.

## 1. Introduction

Both cellular metabolism and external exposures generate reactive oxygen species (ROS) [1,2]. Typically, the body’s antioxidant defense system neutralizes or eliminates ROS [3,4]. However, persistent ROS exposure, due to imbalance in generation and elimination, leads to oxidative stress, causing damage to DNA, proteins, and cells [5,6]. Continuous exposure to oxidative stress is linked to numerous chronic inflammatory conditions, including cardiovascular diseases (CVDs), arthritis, neurodegenerative disorders, and cancers [2,7,8]. Additionally, ROS have been shown to activate nuclear factor kappa B (NF-κB), a key player in the progression of various chronic inflammatory diseases [9,10]. Since ROS-regulated NF-κB activation serves as a crucial mediator of inflammatory responses, inhibiting NF-κB activation is considered an effective approach to treating various chronic inflammatory diseases [11]. Numerous studies have demonstrated that edible and medicinal plants, abundant in bioactive compounds, exhibit enormous potential to protect cells and tissues against oxidative stress and to prevent inflammatory diseases owing to their antioxidative properties [12,13,14,15].

Nuclear factor-erythroid 2-related factor 2 (Nrf2) is a transcription factor pivotal in inducing antioxidative and phase II detoxifying enzyme genes, crucial for exerting antioxidative effects in healthy cells [14,15,16]. Bioactive compounds from edible and medicinal plants exhibit various beneficial effects, including antioxidative and anti-inflammatory activities through activation of Nrf2 and inhibition of NF-κB [17,18,19,20]. Additionally, it has been reported that Nrf2 and NF-κB interact with each other to regulate cellular redox status and coordinate both antioxidant and inflammatory responses [16,21]. However, the underlying mechanism of interaction between the Nrf2 and NF-κB signaling pathways remains unclear, and the association between antioxidative and anti-inflammatory properties remains controversial.

*Polygonum aviculare* L. (PAL), commonly known as knotgrass, belongs to the Polygonaceae family and is grown across many countries in temperate regions [18,20]. PAL is used as a traditional medicine mainly for treating hemorrhoids and pulmonary issues, as well as relieving the symptoms of throat inflammation due to its antimicrobial and anti-inflammatory activities across Asian, African, Latin American and Middle Eastern countries [17,19,22]. Traditionally, it has been therapeutically recommended for treating various inflammatory diseases, including CVDs, rheumatoid arthritis, and bronchitis [23,24,25,26]. PAL has been traditionally used in Korean herbal medicine to treat arthritis and CVDs, where inflammation significantly influences their development and progression [27,28,29]. In vivo studies have shown that PAL treatment suppressed neuroinflammation by reducing tumor necrosis factor-α (TNF-α), interleukin (IL)-1β, and IL-6 production in both the brain and serum from a restraint-stressed model [27,28]. In addition, PAL ameliorated vascular inflammation by reducing NF-κB expression levels in aorta tissue from an Apolipoprotein E knockout model [27,28]. Furthermore, a clinical evaluation of a natural Mexican Sanguinaria extract (PAL) in oral rinse showed a significant decrease in gingivitis, which is an inflammation of the marginal gingiva elicited by bacteria [30]. Several studies have indicated that PAL possesses anti-inflammatory activity owing to a wide variety of secondary metabolites including flavonoids that may be responsible for their antioxidative activity [22,31]. While various studies on the anti-inflammatory and antioxidative activities of PAL have been published, the mechanisms linking these effects remain poorly understood, and no studies have yet reported on this aspect.

This study investigated whether the induction of heme oxygenase-1 (HO-1) via Nrf2 activation is linked to the anti-inflammatory effects of edible and medicinal plants, specifically through modulation of iNOS expression. Additionally, we aimed to establish a crucial link between the antioxidative and anti-inflammatory activities of PAL in RAW 264.7 macrophages. The results showed that PAL effectively induced Nrf2-regulated HO-1 expression, exerting antioxidative effects in RAW 264.7 macrophages. Meanwhile, it suppressed cyclooxygenase-2 (COX-2) and inducible nitric oxide synthase (iNOS) expression through inactivation of NF-κB in lipopolysaccharide (LPS)-activated RAW 264.7 macrophages. Furthermore, these results revealed that Tin protoporphyrin (SnPP), an HO-1 inhibitor, hindered NF-κB inactivation, thereby inhibiting iNOS expression and nitric oxide (NO) generation in LPS-activated RAW 264.7 macrophages treated with the PAL hydroethanolic extract. This study suggests that the interplay of HO-1 serves as a crucial link in the crosstalk between the antioxidative and anti-inflammatory activities of PAL. These findings thus offer insight into a new perspective on how natural plant extracts may ultimately mitigate inflammation through antioxidative responses to oxidative stress.

## 2. Results

### 2.1. PAL Suppresses NF-κB Transcriptional Activity in LPS-Activated RAW 264.7 Macrophages

Since a transcription factor NF-κB serves as a pivotal mediator of inflammatory responses [11], the suppression of NF-κB activity by PAL is deemed an effective therapeutic strategy for various inflammatory diseases, such as neurodegenerative disorders and rheumatoid arthritis.

**Figure 1:**
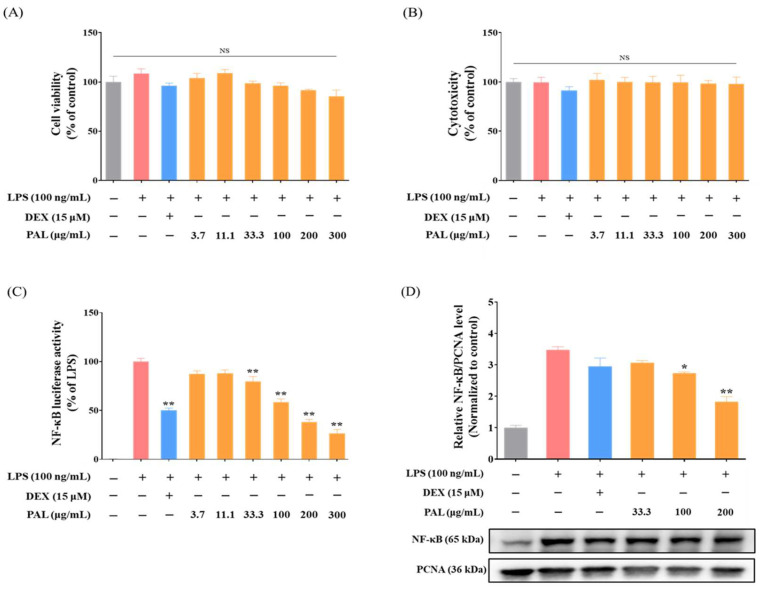
Inhibition of NF-κB activation by PAL hydroethanolic extract. Cell viability of PAL hydroethanolic extract in (**A**) NF-κB Luciferase Reporter-RAW 264.7 cells, and (**B**) RAW 264.7 macrophages was quantified using a CCK-8 assay. (**C**) Concentration-dependent inhibition of NF-κB luciferase activity by PAL hydroethanolic extract in LPS-activated NF-κB Luciferase Reporter-RAW 264.7 cells. (**D**) Expression level of nuclear NF-κB in LPS-activated RAW 264.7 macrophages were quantitatively analyzed. Data are presented as mean  ±  standard error of the mean (SEM) from three independent experiments (*N*  =  3). A statistical significance compared with LPS alone treatment at *p* < 0.05 and *p* < 0.01 was marked by an asterisk (*) and double asterisk (**), respectively. LPS, lipopolysaccharide; DEX, dexamethasone; PAL, *Polygonum aviculare* L.; NS, not significant.

Before examining the anti-inflammatory effects of PAL, cell viability was conducted in both RAW 264.7 macrophages and NF-κB Luciferase Reporter-RAW 264.7 cells to assess whether PAL could cause cytotoxicity. Results showed that PAL did not cause cytotoxicity up to 300 μg/mL in both cell types after 24 h (Figure 1A,B and Figure S1A). Thereafter, PAL concentrations of 33.3, 100, and 200 µg/mL were utilized in the subsequent in vitro experiments.

To explore whether PAL could inhibit NF-κB activity, NF-κB Luciferase Reporter-RAW 264.7 cells were pre-treated with PAL hydroethanolic extract at concentrations of 33.3, 100, and 200 µg/mL for 21 h and co-treated with LPS at 10 ng/mL for 3 h. PAL exhibited a concentration-dependent and significant suppression of NF-κB luciferase activity (Figure 1C). Consistent with the suppression of NF-κB luciferase activity, we observed a concentration-dependent and significant reduction of the nuclear NF-κB expression in LPS-activated RAW 264.7 macrophages in a concentration-dependent fashion (Figure 1D). The findings indicate that NF-κB inhibition by PAL may be potentially useful in preventing and/or treating inflammatory diseases.

### 2.2. PAL Down-Regulates COX-2 and iNOS Expression in LPS-Activated RAW 264.7 Macrophages

**Figure 2:**
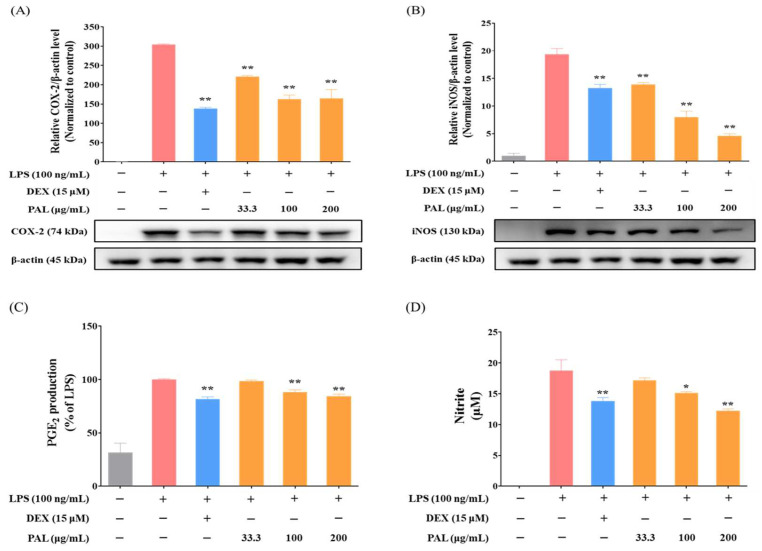
Anti-inflammatory effects of PAL hydroethanolic extract in LPS-activated RAW 264.7 macrophages. Expression levels of (**A**) COX-2 and (**B**) iNOS in LPS-activated RAW 264.7 macrophages were quantitatively analyzed. The levels of extracellular (**C**) PGE_2_ and (**D**) NO were analyzed in LPS-activated RAW 264.7 macrophages. Data are presented as the mean  ±  SEM (*N*  =  3). A statistical significance compared with LPS alone treatment at *p* < 0.05 and *p* < 0.01 was marked by an asterisk (*) and double asterisk (**), respectively.

It is well-known that COX-2 and iNOS are key NF-κB downstream inflammatory mediators that contribute to the inflammation [32,33]. To investigate whether PAL exerts the anti-inflammatory activity by reducing COX-2 and iNOS expression in LPS-activated RAW 264.7 macrophages, Western blotting was conducted. RAW 264.7 macrophages were pre-treated with PAL hydroethanolic extract at concentrations of 33.3, 100, and 200 µg/mL for 3 h, followed by co-treatment with LPS at 100 ng/mL for 21 h. PAL significantly suppressed the elevation of COX-2 and iNOS protein expression levels in LPS-activated RAW 264.7 macrophages (Figure 2A,B). Considering that prostaglandin E_2_ (PGE_2_) and NO production are manipulated by COX-2 and iNOS, respectively, we examined whether PGE_2_ and NO production decreased after PAL pre-treatment in LPS-activated RAW 264.7 macrophages. We observed that PGE_2_ and NO production was significantly suppressed by PAL pre-treatment in LPS-activated RAW 264.7 macrophages in a concentration-dependent fashion (Figure 2C,D). These findings indicate that PAL exerts its anti-inflammatory effects by reducing COX-2 and iNOS expression.

### 2.3. PAL Reduces Pro-Inflammatory Cytokines in LPS-Activated RAW 264.7 Macrophages

**Figure 3:**
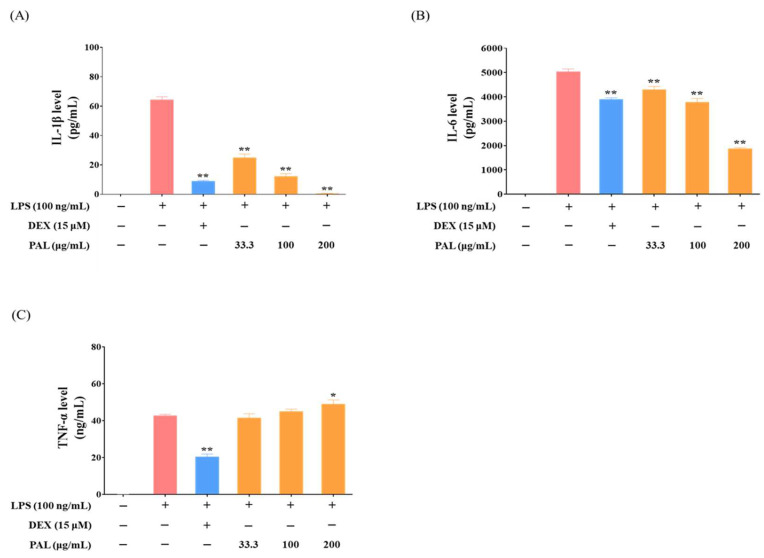
Effects of PAL hydroethanolic extract on the production of pro-inflammatory cytokines in LPS-activated RAW 264.7 macrophages. Cellular inflammatory response was provoked by LPS in RAW 264.7 macrophages. Pro-inflammatory cytokines, including (**A**) IL-1β, (**B**) IL-6, and (**C**) TNF-α were analyzed in LPS-activated RAW 264.7 cells. Data are presented as the mean  ±  SEM (*N*  =  3). A statistical significance compared with LPS alone treatment at *p* < 0.05 and *p* < 0.01 was marked by an asterisk (*) and double asterisk (**), respectively.

Pro-inflammatory cytokines, including IL-1β, IL-6, and TNF-α, are key mediators that contribute to both promoting and regulating inflammation within the body [34]. To examine whether PAL pre-treatment suppresses the elevation of pro-inflammatory cytokine levels in LPS-activated RAW 264.7 macrophages, the levels of IL-1β, IL-6, and TNF-α were determined using ELISA. We observed a concentration-dependent reduction in IL-1β and IL-6 cytokine levels by PAL hydroethanolic extract (Figure 3A,B), while TNF-α levels remained unaffected (Figure 3C). PAL pre-treatment at 200 µg/mL reduced the IL-1β level to approximately 1 pg/mL, and exhibited an almost 40% inhibition of the IL-6 level, compared with that in the LPS treatment (Figure 3A,B). These findings indicate that PAL might exert the anti-inflammatory activity through inhibition of NF-κB.

### 2.4. PAL Induces HO-1 by Nrf2 Activation

**Figure 4:**
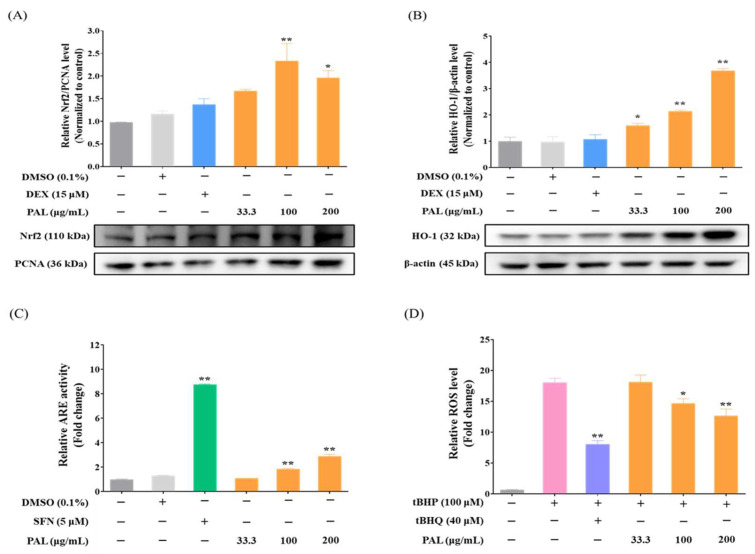
Antioxidant effects of PAL hydroethanolic extract by activation of Nrf2 signaling pathway in RAW 264.7 macrophages. Protein levels of (**A**) nuclear Nrf2 and (**B**) cytoplasmic HO-1 in RAW 264.7 macrophages were quantitatively analyzed. (**C**) ARE activity by PAL hydroethanolic extract in HepG2-ARE cells. (**D**) Intracellular ROS level by PAL hydroethanolic extract in RAW 264.7 macrophages. Data are presented as the mean  ±  SEM (*N*  =  3). A statistical significance compared with control group at *p* < 0.05 and *p* < 0.01 was marked by an asterisk (*) and double asterisk (**), respectively. SFN, sulforaphane; tBHP, tert-butyl hydroperoxide; tBHQ, tertiary-butylhydroquinone.

The Nrf2-mediated induction of HO-1 is one of the intracellular defense responses, thereby maintaining the redox balance against metabolic and oxidative stress [35]. Moreover, this protective process requires the expression of diverse genes that encode proteins playing critical roles in antioxidant/detoxification processes [36]; Nrf2 and HO-1 are key regulators of this process. To examine an Nrf2-mediated induction of HO-1 by PAL, we conducted Western blotting to ascertain whether Nrf2 is translocated into the nucleus, and subsequently cytoplasmic HO-1 expression is elevated by PAL in RAW 264.7 macrophages. We observed a concentration-dependent and significant increase in both nuclear Nrf2 and cytoplasmic HO-1 expression in PAL-treated RAW 264.7 macrophages (Figure 4A,B). Furthermore, Nrf2 nuclear translocation induces the expression of HO-1 and phase II detoxifying enzymes via ARE in the promoters of those genes [37]. To evaluate whether PAL induces ARE activity, cell viability and ARE luciferase activity assays were performed in HepG2-ARE cells. Cell viability assay showed that PAL was not cytotoxic at any of the tested concentrations in HepG2-ARE cells (Figure S1B). Moreover, PAL exhibited a concentration-dependent increase in ARE activity within the cells (Figure 4C).

Considering that PAL possesses antioxidative activity through the Nrf2-mediated induction of HO-1, we evaluated whether PAL pre-treatment suppresses ROS generation in RAW 264.7 macrophages using a DCFDA assay. We observed that PAL pre-treatment significantly inhibited ROS production in RAW 264.7 macrophages treated with tBHQ, an ROS inducer (Figure 4D), suggesting that HO-1 induction is a prerequisite for the suppression of ROS production. These results indicate that the PAL-induced reduction of ROS generation in RAW 264.7 macrophages was a consequence of the Nrf2-mediated induction of HO-1.

### 2.5. PAL Suppresses iNOS Expression by Inducing HO-1

**Figure 5:**
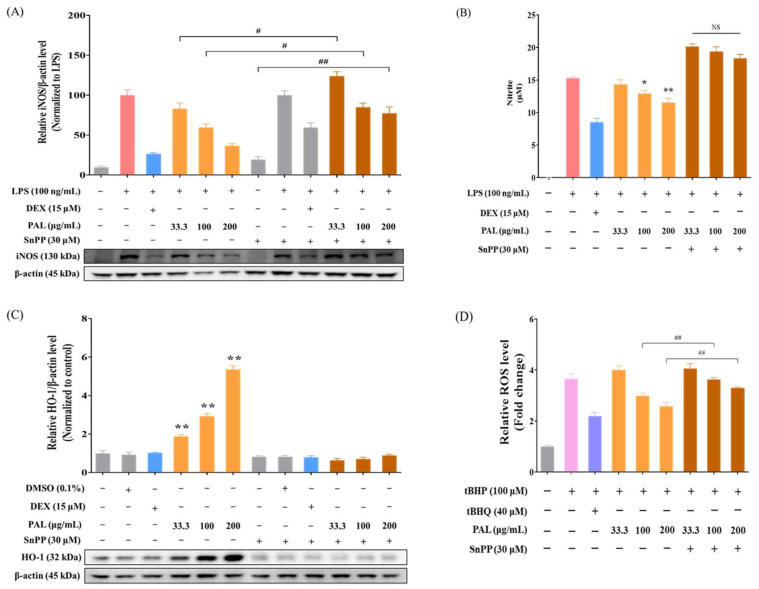
HO-1 inhibition nullified anti-inflammatory effects by PAL hydroethanolic extract. (**A**) Cytoplasmic iNOS protein level was quantitatively analyzed in RAW 264.7 cells pre-treated with or without SnPP. (**B**) Extracellular NO level by PAL hydroethanolic extract in RAW 264.7 cells pre-treated with or without SnPP. (**C**) Cytoplasmic HO-1 protein level was quantitatively analyzed in RAW 264.7 cells pre-treated with or without SnPP. (**D**) Intracellular ROS level was analyzed in LPS-activated RAW 264.7 cells pre-treated with and without SnPP. Values are mean  ±  SEM (*N*  =  3). A significance difference compared with LPS alone or control group at *p* < 0.05 and *p* < 0.01 was indicated by an asterisk (*) and double asterisk (**), respectively. A hash (# *p* < 0.05) and double hash (## *p* < 0.01) indicate a significant difference between groups. SnPP, Tin Protoporphyrin IX dichloride.

Numerous studies have reported a close connection between antioxidative and anti-inflammatory activities. Sustained exposure to enhanced ROS production can trigger inflammatory responses through the activation of NF-κB and subsequent induction of its downstream mediators [9]. Several studies have reported that inducing HO-1 expression is closely connected to enhanced removal of ROS and the maintenance of intracellular redox balance [38]. To explore whether HO-1 induction is involved in the anti-inflammatory activity of PAL, we conducted Western blotting and Griess assay in RAW 264.7 macrophages pre-treated with SnPP, an HO-1-specific inhibitor, aiming to determine iNOS expression and NO production levels. HO-1 inhibitor showed no cytotoxicity at the tested concentrations (Figure S1C). We observed that the HO-1 inhibitor hindered the reduction of both iNOS expression and NO generation by PAL in LPS-activated RAW 264.7 cells (Figure 5A,B). Furthermore, we found that the HO-1 inhibitor interfered with the decrease of NF-κB luciferase activity by PAL in LPS-activated NF-κB Luciferase Reporter-RAW 264.7 cells (Figure S3A). In addition, our results revealed that HO-1 expression was not elevated by PAL in RAW 264.7 cells pre-treated with the HO-1 inhibitor (Figure 5C). Consequently, ROS levels were significantly higher in RAW 264.7 cells pre-treated with the HO-1 inhibitor compared to those pre-treated without the HO-1 inhibitor (Figure 5D). This established that PAL exerts its anti-inflammatory activity by inducing HO-1 expression and suppressing iNOS expression.

### 2.6. Seven Phytochemicals Were Detected in PAL

Liquid chromatography-mass spectrometry (LC-MS) methods are extensively employed for the identification and characterization of phytochemical components of diverse traditional herbal medicines and their associated formulations [39]. In particular, the ultra-high-performance LC (UPLC)-tandem mass spectrometry (MS/MS) method using an Orbitrap analyzer is widely utilized because of low mass error owing to high sensitivity and resolution [40]. In this study, PAL hydroethanolic extract was analyzed using the UPLC-MS/MS, to identify the major phytochemicals present. We identified seven phytochemicals based on the retention time and mass spectra of reference standard compounds and published studies [41]. These included digalloylhexoside, myricitrin, avicularin, quercitrin, myricetin, quercetin (QUE), and kaempferol (KAE) (Table 1). Figure S2A,B illustrates the base peak ion chromatograms of PAL hydroethanolic extract on positive ion mode and extracted ion chromatograms of compounds detected in PAL hydroethanolic extract, respectively. Additionally, we observed that KAE and QUE significantly suppressed NF-κB luciferase activity in LPS-activated NF-κB Luciferase Reporter-RAW 264.7 cells, compared to other compounds detected in PAL hydroethanolic extract (Figure S3B). These findings suggest that KAE and QUE are the main components contributing to the anti-inflammatory effects of PAL.

### 2.7. Inhibition of HO-1 Expression Nullified the Reduction in NF-κB Activity and NO Production Induced by KAE and QUE

**Figure 6:**
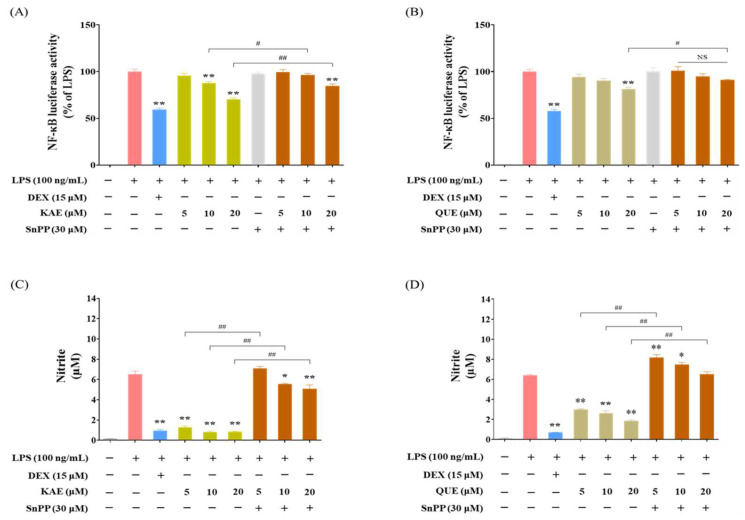
HO-1 inhibition abrogates anti-inflammatory effects by KAE and QUE abundant in PAL hydroethanolic extract. NF-κB luciferase activity by (**A**) KAE and (**B**) QUE in LPS-activated NF-κB Luciferase Reporter-RAW 264.7 cells pre-treated with or without SnPP. Extracellular NO level by (**C**) KAE and (**D**) QUE in LPS-activated RAW 264.7 macrophages pre-treated with and without SnPP. Data are presented as the mean  ±  SEM (*N*  =  3). A statistical significance compared with LPS alone group at *p* < 0.05 and *p* < 0.01 was marked by an asterisk (*) and double asterisk (**), respectively. A hash (# *p* < 0.05) and double hash (## *p* < 0.01) indicate a statistical significance between groups. NS, not significant.

Given that KAE and QUE are the major bioactive compounds in PAL hydroethanolic extract, the anti-inflammatory activity exhibited by the PAL hydroethanolic extract is attributed to these two components. To investigate whether the anti-inflammatory activity of PAL hydroethanolic extracts involves KAE and QUE, we conducted NF-κB luciferase and Griess assays in RAW 264.7 macrophages pre-treated with and without the HO-1 inhibitor to determine NF-κB activity and NO production. We observed that the HO-1 inhibitor hindered the reduction of NF-κB activation induced by KAE and QUE in LPS-activated RAW 264.7 macrophages (Figure 6A,B). Additionally, the reduction of NO production was nullified by the HO-1 inhibitor in LPS-activated RAW 264.7 macrophages treated with KAE and QUE (Figure 6C,D). These findings imply that KAE and QUE abundant in the PAL hydroethanolic extract exhibit anti-inflammatory activity by inducing HO-1 and inhibiting iNOS.

## 3. Discussion

An imbalance in redox caused by increased ROS production triggers inflammatory diseases, including arthritis, neurodegenerative disorders, and cancer, through the NF-κB activation [9,42,43,44,45]. Therefore, suppression of NF-κB activity may reduce the risk of inflammatory diseases by protecting healthy cells from oxidative stress. Edible medicinal plants containing various bioactive compounds have been found to directly or indirectly induce antioxidative and phase II detoxifying enzymes, which play crucial roles in exerting anti-inflammatory effects [15,46,47,48]. However, the underlying mechanisms linking the antioxidative and anti-inflammatory properties in these edible medicinal plants remain unclear. In this study, we explored whether the antioxidative and anti-inflammatory effects exhibited by the PAL hydroethanolic extract in RAW 264.7 macrophages were linked via an intracellular mechanism.

NF-κB is a well-known transcription factor that plays a pivotal role in inflammatory responses and is implicated in various inflammatory diseases [11,49]. In this study, the PAL hydroethanolic extract significantly inhibited NF-κB activation in LPS-activated RAW 264.7 macrophages (Figure 1C). Numerous studies have addressed the detrimental effects associated with the production of specific pro-inflammatory cytokines, including IL-1β, IL-6, and TNF-α, which act as the progression of inflammatory diseases [50,51,52]. We observed that the PAL hydroethanolic extract significantly inhibited IL-1β and IL-6 levels in LPS-activated RAW 264.7 macrophages (Figure 3A,B). Additionally, COX-2 and iNOS, known for promoting the inflammatory mediators PGE_2_ and NO, respectively, are recognized for their role in causing inflammation and tissue damage [53,54]. We observed that the PAL hydroethanolic extract suppressed NO production only, although it also decreased both COX-2 and iNOS expressions (Figure 2A–D). Furthermore, the HO-1 inhibitor nullified suppression of iNOS expression and NO production as well as NF-κB activity by the PAL hydroethanolic extract (Figure 5A,B and Figure S3A). These findings indicate that the anti-inflammatory activity exhibited by the PAL hydroethanolic extract was regulated by inducing HO-1 expression and inhibiting iNOS/NO signaling.

KAE and QUE, abundant in PAL, are well-known for their antioxidative and anti-inflammatory activities [55,56]. In particular, kaempferol-3-O-β-d-glucuronate (K3G) has been reported to exert antioxidative and anti-inflammatory activities by activating Nrf2/HO-1 signaling and inhibiting mitogen-activated protein kinases/NF-κB signaling [57]. Furthermore, it has been reported that K3G down-regulated proinflammatory cytokines, such as IL-1β, PGE_2_, and NO by suppressing NF-κB both in vitro and in vivo, while it up-regulated anti-inflammatory cytokine IL-10 [58]. Given that KAE and QUE have inhibited NF-κB activation and subsequently reduced NO production (Figure 6A–D), the anti-inflammatory effect exhibited by PAL can be attributed to KAE and/or QUE.

Numerous studies have shown that anti-inflammatory and antioxidative activities are closely connected [17,18,19,20]. The Nrf2 activation, which leads to the accumulation of glutathione, shifts the redox balance in cells towards a more reducing environment and effectively eliminates ROS, potentially inhibiting NF-κB activation [21,59]. Aside from the activation of the Nrf2, the interaction between Kelch-like ECH-associated protein 1 and the IκappaB kinase (IKK)β subunit of the IKK complex has been found to negatively regulate NF-κB activation through the stabilization of IKBα [21,60].

Considering that HO-1 end-products, including carbon monoxide, act as an antioxidant by scavenging or neutralizing ROS and inhibiting iNOS that promotes the generation of pro-inflammatory cytokines [61], we speculated that a negative interaction between the HO-1 and iNOS would ameliorate inflammatory responses [62,63,64]. In this study, we observed that the HO-1 inhibitor SnPP significantly negated the inhibition of NF-κB activity, iNOS expression, and NO production by PAL, compared with those in LPS-activated RAW 264.7 macrophages without SnPP. Collectively, these findings highlight that the induction of Nrf2-mediated HO-1 expression by PAL is crucial for NF-κB inactivation, exerting an anti-inflammatory effect by reducing iNOS expression levels, and in turn inhibiting NO production. However, further research is required to understand linking the antioxidative and anti-inflammatory properties exhibited by medicinal herbs and to provide new insights to elucidate the mechanisms involved in the interplay between the Nrf2 and NF-κB.

## 4. Materials and Methods

### 4.1. Chemicals

Kaempferol (KAE) and quercetin (QUE) were purchased from ChemFaces (Wuhan, China). SnPP (Santa Cruz Biotechnology, Dallas, TX, USA) was obtained from Sigma-Aldrich, Inc. (St. Louis, MO, USA). All other chemicals were obtained from Sigma-Aldrich, Inc. unless otherwise stated.

### 4.2. Preparation of Hydroethanolic Extract of PAL

PAL was obtained from the National Institute for Korean Medicine Development (Gyeongsan, South Korea). Five hundred grams of dried PAL were subjected to heat reflux extraction in 3.5 L of 70% ethanol for 3 h. The hydroethanolic extract of PAL was filtered using Cytiva Whatman™ Qualitative Filter Papers—Grade 2V (Whatman plc, Little Chalfont, Buckinghamshire, UK), and then lyophilized using a Table Top Freeze Dryer (Ilshin Biobase, Dongducheon, Republic of Korea). The lyophilized powder of PAL hydroethanolic extract was kept at −20 °C until further use. A voucher specimen (#JE-176) has been deposited in the herbarium of the KIOM (Daejeon, Republic of Korea).

### 4.3. Cell Lines and Culture

RAW 264.7 murine macrophage cells were passaged in Dulbecco’s modified Eagle medium (DMEM; #11995-065; Thermo Fisher Scientific Inc., Waltham, MA, USA) containing 10% (*v*/*v*) fetal bovine serum (FBS; #16000-044; Thermo Fisher Scientific Inc.) and 1% (*v*/*v*) Penicillin/Streptomycin (Pen/Strep) (#15140122, Thermo Fisher Scientific Inc.).

NF-κB Luciferase Reporter RAW 264.7 cells stably expressing firefly luciferase containing the NF-κB response element sequence was purchased from BPS Bioscience (#79978, San Diego, CA, USA). Cells were passaged in DMEM containing 10% (*v*/*v*) FBS, 1% (*v*/*v*) Pen/Strep, 1% (*v*/*v*) GlutaMax (#35050061, Thermo Fisher Scientific Inc.) and Geneticin™ Selective Antibiotic (G418 Sulfate, #10131-027, Thermo Fisher Scientific Inc., 700 μg/mL). Transfected cells were clonally selected by exposure to Geneticin.

HepG2 human hepatic cells harboring the construct containing the antioxidant response element (ARE) sequence following the luciferase-encoding gene were purchased from BPS Bioscience (#60513). Cells were passaged in MEM containing 8% (*v*/*v*) FBS, 1% (*v*/*v*) Pen/Strep, and Geneticin™ Selective Antibiotic (G418 Sulfate, #10131-027, Gibco Thermo Fisher Scientific Inc., 600 μg/mL). Transfected cells were clonally selected by exposure to Geneticin.

All cells were maintained in a humidified CO_2_ incubator (HERACELL 150i; Gibco Thermo Fisher Scientific Inc.) at 37 °C and 5% CO_2_. The cells were sub-cultured when they reach to approximately 90% confluency.

### 4.4. NF-κB Luciferase Activity Assay

To assess the NF-κB transcriptional activity, an NF-κB luciferase activity assay was performed in NF-κB Luciferase Reporter RAW 264.7 cells using a luciferase assay system (Promega, Madison, WI, USA) following the manufacturer’s instructions with modification. The cells were seeded in a 96-well plate at 4 × 10^4^ cells per well. After 24 h of incubation, the cells were pre-treated with the hydroethanolic extract of PAL for 21 h and co-treated with lipopolysaccharide (LPS, Sigma-Aldrich, Inc.) at 10 ng/mL for additional 3 h. The NF-κB luciferase activity was assessed by quantifying the amount of luminescence using a TriStar LB 941 multimode microplate reader (Berthold Technologies GmbH & Co.KG, Bad Wildbad, Germany). The luminescence was then normalized against the control. Dexamethasone (DEX, 15 μM; Sigma-Aldrich, Inc.) was used as a positive control.

### 4.5. Determination of NO Production (Griess Assay)

RAW 264.7 cells were seeded in a 96-well plate at 6 × 10^4^ cells per well. After 24 h of incubation, the cells were pre-treated with the PAL hydroethanolic extract for 3 h, and then co-treated with LPS at 100 ng/mL. After 24 h, the culture medium was harvested. The NO level in the culture medium was assessed by indirectly quantifying the stable NO catabolite, nitrite, using the Griess assay. DEX (15 μM) was used as a positive control.

### 4.6. Enzyme-Linked Immunosorbent Assay (ELISA)

Pro-inflammatory cytokine levels in the culture medium were measured for IL-1β, IL-6, and TNF-α using commercial ELISA kits (R&D Systems, Inc., Minneapolis, MN, USA). The culture medium was subjected to ELISA for each cytokine, following the manufacturer’s instructions. DEX (15 μM) was used as a positive control.

### 4.7. ARE-Luciferase Activty Assay

To determine ARE transcriptional activity, ARE luciferase activity assay was performed in HepG2-ARE cells as previously described [65]. Briefly, the cells were seeded in a 96-well plate at 2.5 × 10^4^ cells per well. After 24 h incubation, the cells were treated with the hydroethanolic extract of PAL for an additional 24 h. ARE luciferase activity was assessed by quantifying the amount of luminescence using a TriStar LB 941 multimode microplate reader (Berthold Technologies GmbH & Co.KG). The luminescence was then normalized against the control. Sulforaphane (SFN, #S4441, Sigma-Aldrich, Inc., 5 μM) was used as a positive control [66].

### 4.8. Measurement of Intracellular ROS Level

The ROS level was determined by a commercial cellular ROS Assay kit (#ab113851, abcam, Boston, MA, USA) with modification. RAW 264.7 cells were seeded in a 96-well black plate with a clear bottom (SPL Life Sciences Co., Ltd.) at 2.5 × 10^4^ cells per well. After 24 h of incubation, the cells were treated with the PAL hydroethanolic extract for an additional 24 h. Cells were then pre-incubated with the cell-permeant reagent 20 μM 2′,7′-dichlorodihydrofluorescein diacetate (H_2_DCFDA) to quantitatively assess fluorescent 2′,7′-dichlorofluorescein (DCF) by ROS for 30 min. Subsequently, the cells were subjected to tert-butyl hydroperoxide (tBHP, a membrane-permeant pro-oxidant; 100 μM in 1% FBS-containing PBS) for 1 h. Fluorescence was quantified at excitation and emission wavelengths of 485 nm and 535 nm, respectively, using a BioTek Synergy HTX microplate reader (BioTek Instruments, Inc.). Tert-butylhydroquinone (tBHQ, Sigma-Aldrich, Inc., 40 μM) was used as a positive control.

### 4.9. Statistical Analysis

Comparisons among the averaged values were performed using one-way analysis of variance, followed by Student’s *t*-test and Dunnett’s test, with a significance level at *p* < 0.05 and *p* < 0.01. Statistical analyses were carried out using GraphPad Prism 9.0 (GraphPad Software, San Diego, CA, USA), and statistical significance in graphs are marked using an asterisk (*) or hash (#) in the graphs.

## 5. Conclusions

In conclusion, we demonstrated that the hydroethanolic extract of PAL inhibited NF-κB activation, thereby reducing the levels of pro-inflammatory cytokines and iNOS-mediated inflammatory processes. Furthermore, we observed that inhibiting HO-1 induction abolished NF-κB inactivation by the hydroethanolic extract of PAL and subsequently elevated the levels of NO production mediated by iNOS in LPS-activated RAW 264.7 macrophages. However, further studies are required to clarify the current findings regarding the link between anti-inflammatory and antioxidative activities of PAL and to elucidate whether bioactive compounds may be responsible for mediating the interplay between the Nrf2 and NF-κB signaling pathways. Collectively, we demonstrated a reciprocal inhibition between the Nrf2 and NF-κB signaling pathways, suggesting that the antioxidative and anti-inflammatory effects of PAL hydroethanolic extract may be closely linked by the inhibition of HO-1-mediated iNOS.

## Figures and Tables

**Table 1:** Phytochemical components of PAL hydroethanolic extract by UPLC-MS/MS.

No	R_t_ (min)	Calculated (*m*/*z*)	Measured (*m*/*z*)	Error (ppm)	Adduct	Formula	MS/MS Fragments (*m*/*z*)	Identification
1	4.93	483.0780	483.0789	1.804	[M-H]^−^	C_20_H_20_O_14_	331, 313, 271	Digalloylhexoside [23]
2	6.68	463.0882	463.0891	1.968	[M-H]^−^	C_21_H_20_O_12_	-	Myricetrin *
3	7.31	433.0776	433.0781	1.143	[M-H]^−^	C_20_H_18_O_11_	-	Avicularin *
4	7.54	447.0933	447.0939	1.324	[M-H]^−^	C_21_H_20_O_11_	447, 301	Quercitrin *
5	8.05	317.0303	317.0305	0.718	[M-H]^−^	C_15_H_10_O_8_	-	Myricetin *
6	9.59	301.0354	301.0359	1.588	[M-H]^−^	C_15_H_10_O_7_	-	Quercetin *
7	11.05	285.0405	285.041	1.842	[M-H]^−^	C_15_H_10_O_6_	-	Kaempferol *

* compared with retention time and mass spectrum of reference standards. R_t_, retention time.

## Data Availability

Data is contained within the article and Supplementary Materials.

## Supplementary Materials

The following supporting information can be downloaded at: https://www.mdpi.com/article/10.3390/plants13233314/s1, Figure S1. Cell viability by PAL hydroethanolic extract; Figure S2. UPLC-MS/MS analysis of PAL hydroethanolic extract; Figure S3. NF-κB luciferase activity by the major phytochemicals in PAL hydroethanolic extract; Figure S4. The original Western blotting images. Reference [67] is cited in the Supplementary Materials.
