# Supplementary material for: Hydroethanolic Extract of Polygonum aviculare L. Mediates the Anti-Inflammatory Activity in RAW 264.7 Murine Macrophages Through Induction of Heme Oxygenase-1 and Inhibition of Inducible Nitric Oxide Synthase
Source: Plants (Basel). 2024 Nov 26;13(23):3314. doi: 10.3390/plants13233314 (PMC11644326; doi:10.3390/plants13233314)
Supplement: Supplementary file 1 [file plants-13-03314-s001.zip › plants-3236600-supplementary.pdf]

Supplementary Materials and Methods

Cell viability assay

RAW 264.7 cells were seeded in a 96-well plate (SPL Life Sciences Co. Ltd.) at 6 × 10^4 cells per well. After 24 h of incubation, the cells were treated with hydroethanolic extract of PAL (3.7, 11.1, 33,3, 100, 200, and 300 μg/mL), KAE (0–20 μM) and QUE (0–20 μM). After 24 h of treatment, the medium was discarded, and then 110 μL of diluted cell counting kit-8 (CCK-8) solution (Dojindo Laboratories, Kumamoto, Japan) in phosphate-buffered saline (PBS) were added to each well. Relative cell viability was assessed by quantifying the amount of formazan dye produced via intracellular dehydrogenase enzymatic activity, directly proportional to the number of living cells in each well, at an absorbance of 450 nm using a BioTek Synergy HTX microplate reader (BioTek Instruments, Inc., Winooski, VT, USA).

Western blotting

RAW 264.7 cells were seeded in a 6-well plate at a density of 6 × 10^5 cells per well. After 24 h of incubation, the cells were pre-treated with the PAL hydroethanolic extract for 3 h, and then co-treated with LPS at 100 ng/mL. After 24 h, cells were then harvested. The cells were lyzed using the NE-PER™ Nuclear and Cytoplasmic Extraction Reagents (#78835; Thermo Fisher Scientific Inc.) containing 1X protease inhibitor (#78444; Thermo Fisher Scientific Inc.) following the manufacturer's instructions. Protein quantification was conducted in both nuclear and cytoplasmic fractions using the Pierce™ bicinchoninic acid protein assay kit (#23225, Thermo Fisher Scientific Inc.). Equal quantities of nuclear and cytoplasmic proteins were separated on the 10% Mini-PROTEAN® TGX™ Precast Protein Gels (#4561036; Bio-Rad, San Francisco, CA, USA) and subsequently transferred to the Trans-Blot Turbo Mini 0.2 μm polyvinylidene difluoride membrane (#1704156; Bio-Rad). Subsequently, the membrane was blocked in blocking solution (#37527; Thermo Fisher Scientific Inc.) and sequentially incubated with primary and secondary antibodies. The primary antibodies were immunoglobulin G (IgG) against Nrf2 (#ab137550; Abcam), HO-1 (#ab13243; Abcam), COX-2 (#12282; Cell Signaling Technology), iNOS (#2982; Cell Signaling Technology), NF-κB (#8242; Cell Signaling Technology), proliferating cell nuclear antigen (PCNA, #13110, Cell Signaling Technology), and β-actin (#4967, Cell Signaling Technology) at a dilution of 1:1,000. Horseradish peroxidase-conjugated secondary antibodies were anti-rabbit IgG (#7074; Cell Signaling Technology) and anti-mouse IgG (#7076; Cell Signaling Technology) at a dilution of 1:5,000. The antibody-bound protein bands were detected using the SuperSignal™ West Femto Maximum Sensitivity Substrate kits (#34095; Thermo Fisher Scientific Inc.) and visualized using a ChemiDoc™ Touch Imaging System (Bio-Rad). The detected bands were densitometrically analyzed using Image Lab (Bio-Rad).

Phytochemical profiling of PAL using ultra-high-performance liquid chromatography-tandem mass spectrometry (UPLC-MS/MS)

Phytochemical components in hydroethanolic extract of PAL were identified by comparing their retention time and mass spectrum with those of reference standards and reported data [1]. Reference standards, including myricetrin, avicularin, quercitrin, myricetin, quercetin, and kaempferol, were obtained from TargetMol (Wellesley Hills, MA, USA). The freeze-dried hydroethanolic extract of PAL dissolved in 100% methanol was analyzed using a Dionex UltiMate 3000 system coupled with a Thermo Q-Exactive mass spectrometer (Thermo Fisher Scientific). The gradient eluent conditions for UPLC-MS/MS analysis were employed as previously described [2]. Briefly, chromatographic separation was performed on an Acquity BEH C18 column (100 × 2.1 mm,

1.7 µm) with the optimal mobile phase composed of 0.1% (v/v) formic acid in water and acetonitrile. Data acquisition and processing were performed using Xcalibur and TraceFinder 5.1 softwares (Thermo Fisher Scientific Inc.).

References

1. Pawłowska, K.A.; Kryżman, M.; Zidorn, C.; Pagitz, K.; Popowski, D.; Granica, S. HPLC-DAD-MS3 fingerprints of phenolics of selected Polygonum taxa and their chemometric analysis. *Phytochemistry* **2023**, *208*, 113605, doi:10.1016/j.phytochem.2023.113605.
2. Hwang, Y.H.; Jang, S.A.; Kim, T.; Ha, H. Anti-osteoporotic and Anti-adipogenic Effects of Rhus chinensis Nutgalls in Ovariectomized Mice Fed with a High-fat Diet. *Planta. Med.* **2019**, *85*, 1128–1135, doi:10.1055/a-0989-2585.

(A)

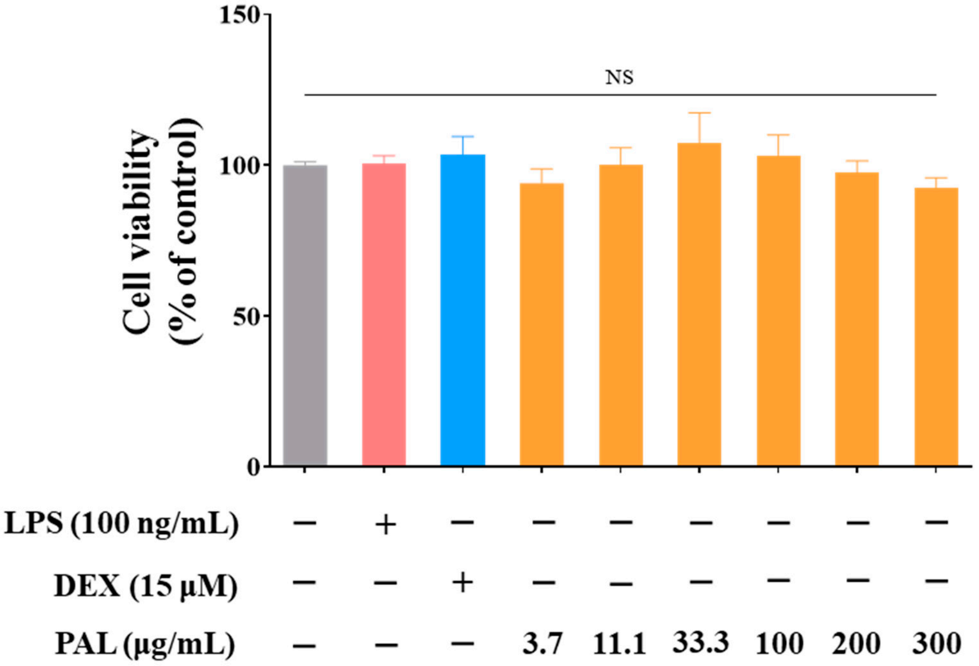

(B)

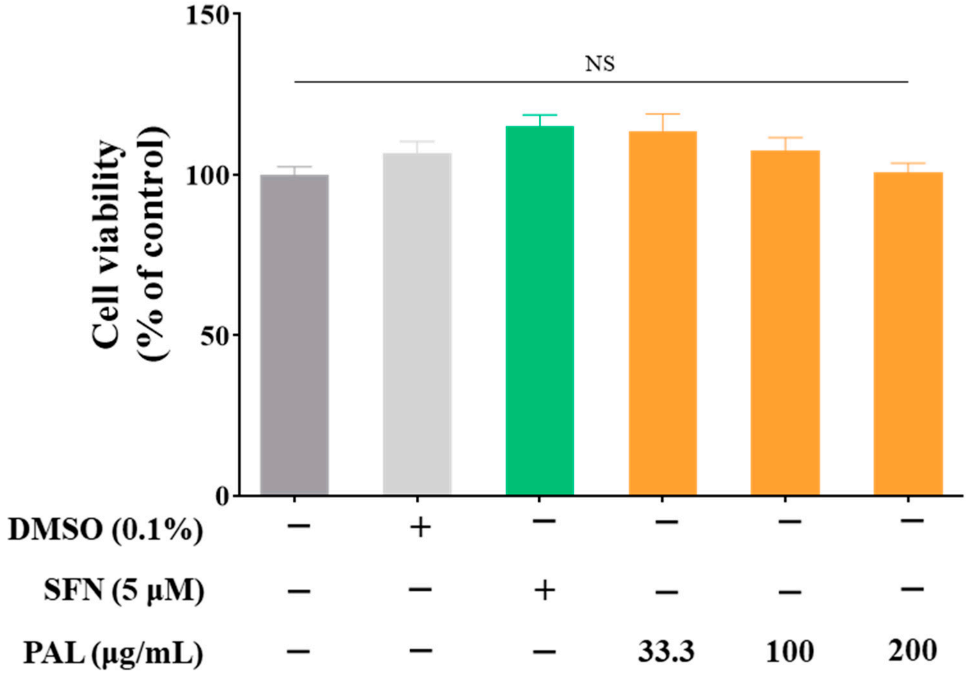

(C)

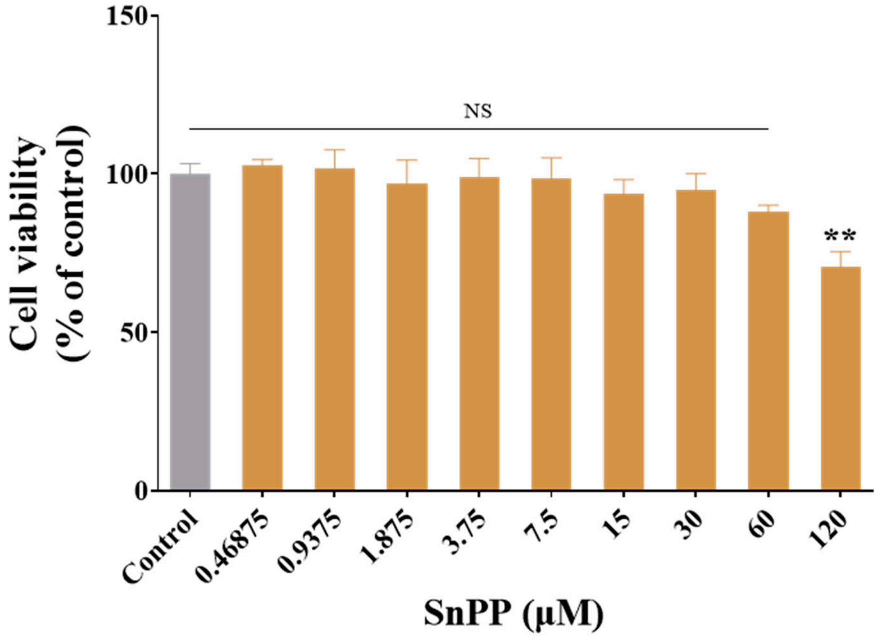

Figure S1. Cell viability by PAL hydroethanolic extract. (A) Cell viability by PAL hydroethanolic extract in RAW 264.7 macrophages was quantified using a CCK-8 assay. (B) Cell viability by PAL hydroethanolic extract in HepG2-ARE cells was quantified using a CCK-8 assay. (C) Cell viability by SnPP in RAW 264.7 macrophages was quantified using a CCK-8 assay. Data are expressed as mean ± SEM from three independent experimental sets (*N* = 3). A statistical significance compared with LPS

alone or control group at $p < 0.05$ and $p < 0.01$ was marked by an asterisk (*) and double asterisk (**), respectively. NS, not significant.

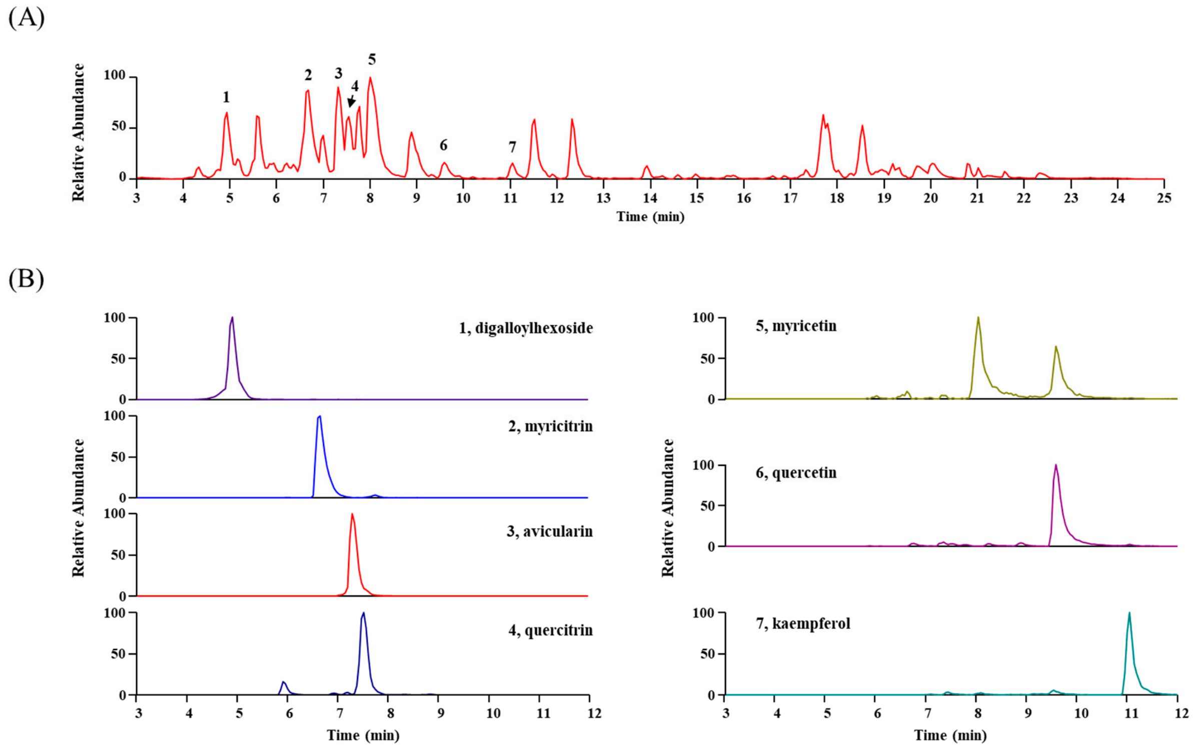

Figure S2. UPLC-MS/MS analysis of PAL hydroethanolic extract. (A) Base peak ion chromatograms of PAL with positive ionization mode on UPLC-MS/MS. (B) Extracted ion chromatograms of identified phytochemicals with retention time (min) between 3 and 14 min. 1, digalloylhexoside; 2, myricitrin; 3, avicularin; 4, quercitrin; 5, myricetin; 6, quercetin; 7, kaempferol.

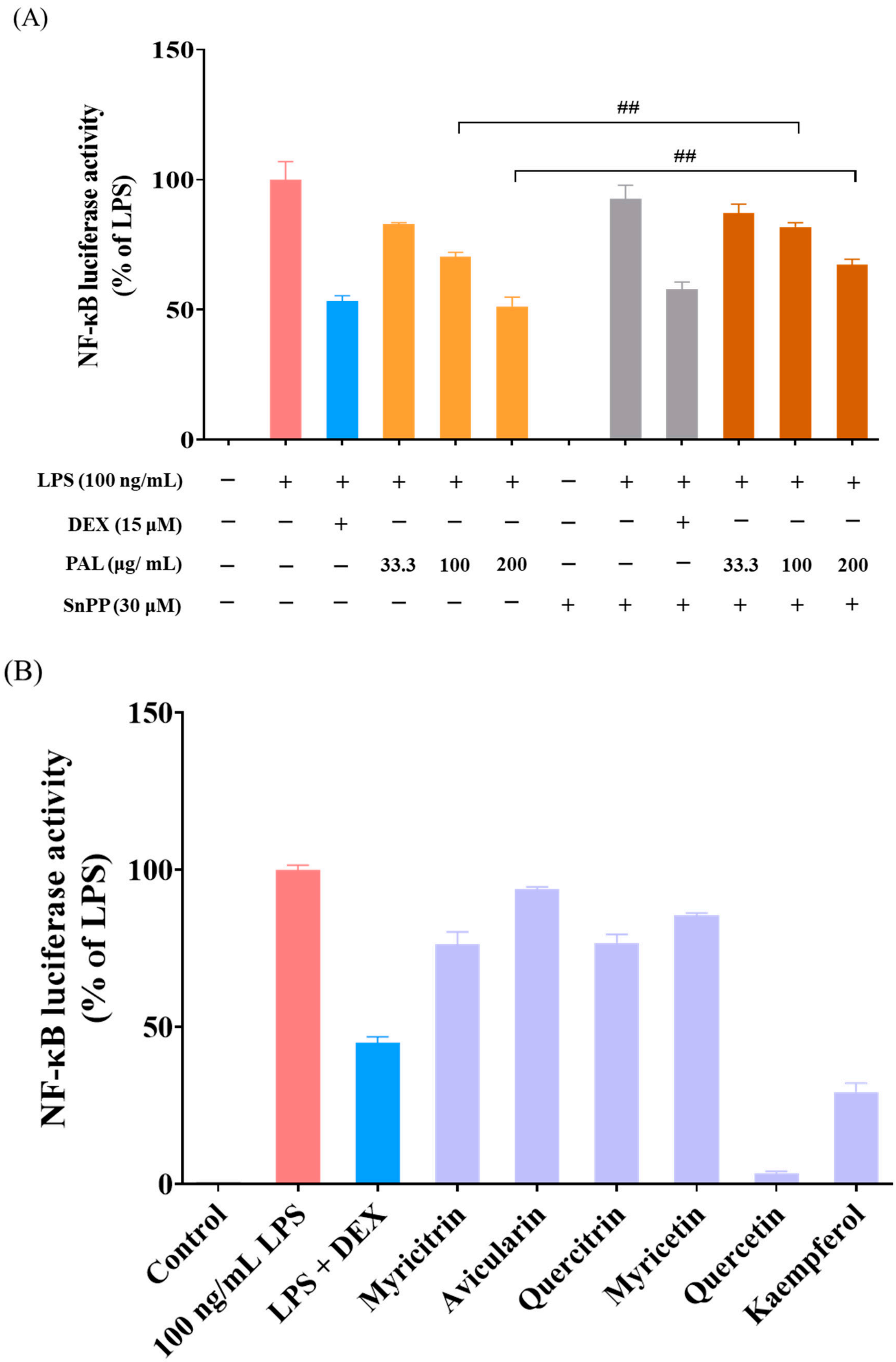

Figure S3. NF-κB luciferase activity by the major phytochemicals in PAL hydroethanolic extract. (A) NF-κB activity by PAL hydroethanolic extract in LPS-activated NF-κB Luciferase Reporter-RAW 264.7 cells pre-treated with or without SnPP. Values are mean ± SEM ($N = 3$). A hash (# $p < 0.05$) and double

hash (## $p < 0.01$) indicate a significant difference between groups. (B) NF-κB luciferase activity by individual phytochemical at 20 μM identified in PAL hydroethanolic extract in LPS-activated NF-kB Luciferase Reporter-RAW 264.7 cells. Data are expressed as mean ± SD ($n = 2$). SnPP, Tin Protoporphyrin IX dichloride.

(A)

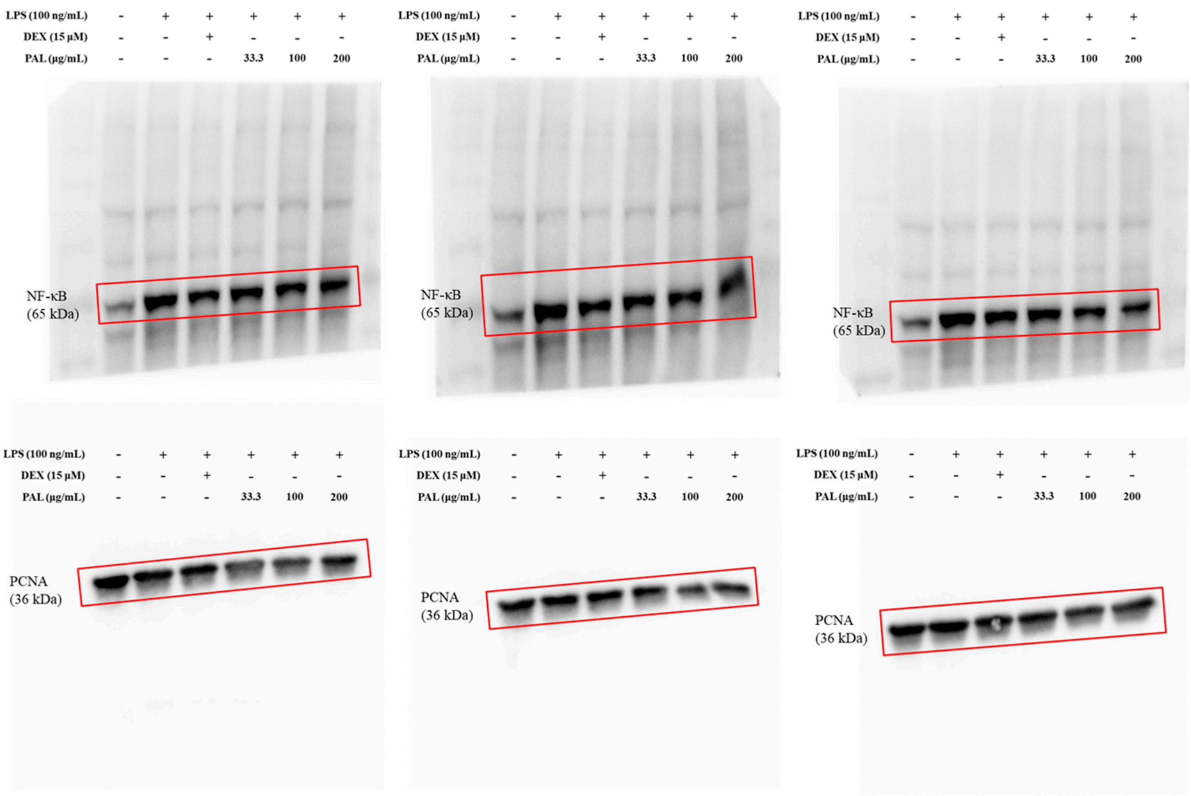

(B)

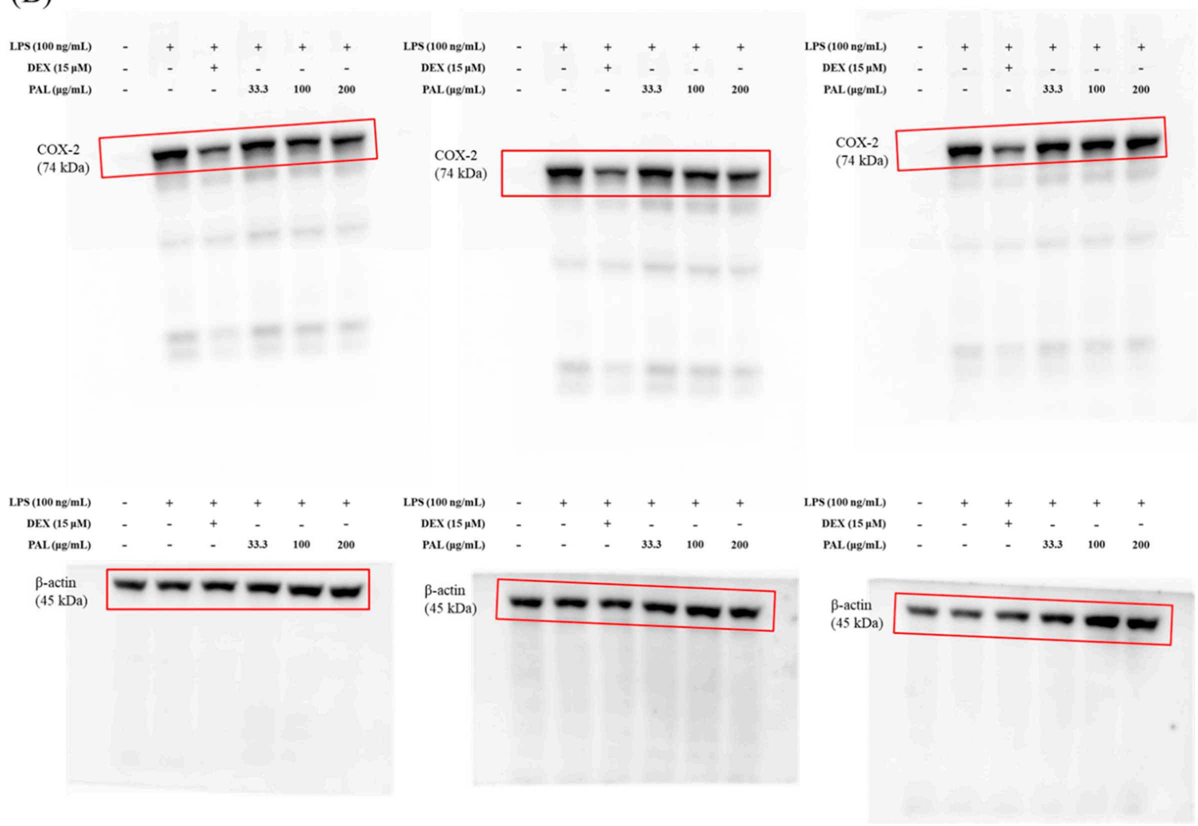
LPS (100 ng/mL) - + + + + +
DEX (15 μM) - - + - - -
PAL (μg/mL) - - - 33.3 100 200
COX-2 (74 kDa)
β-actin (45 kDa)

(C)

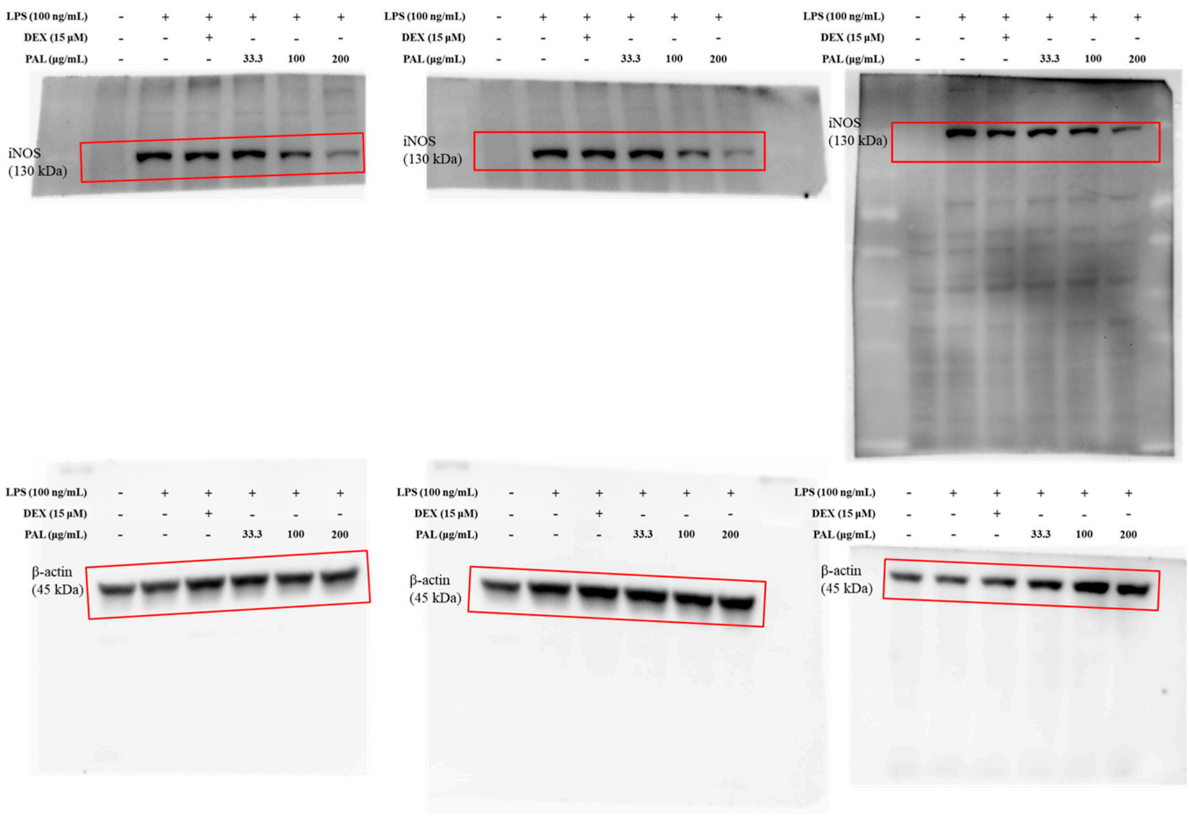
LPS (100 ng/mL) - + + + + +
DEX (15 μM) - - + - - -
PAL (μg/mL) - - - 33.3 100 200
iNOS (130 kDa)
β-actin (45 kDa)

(D)

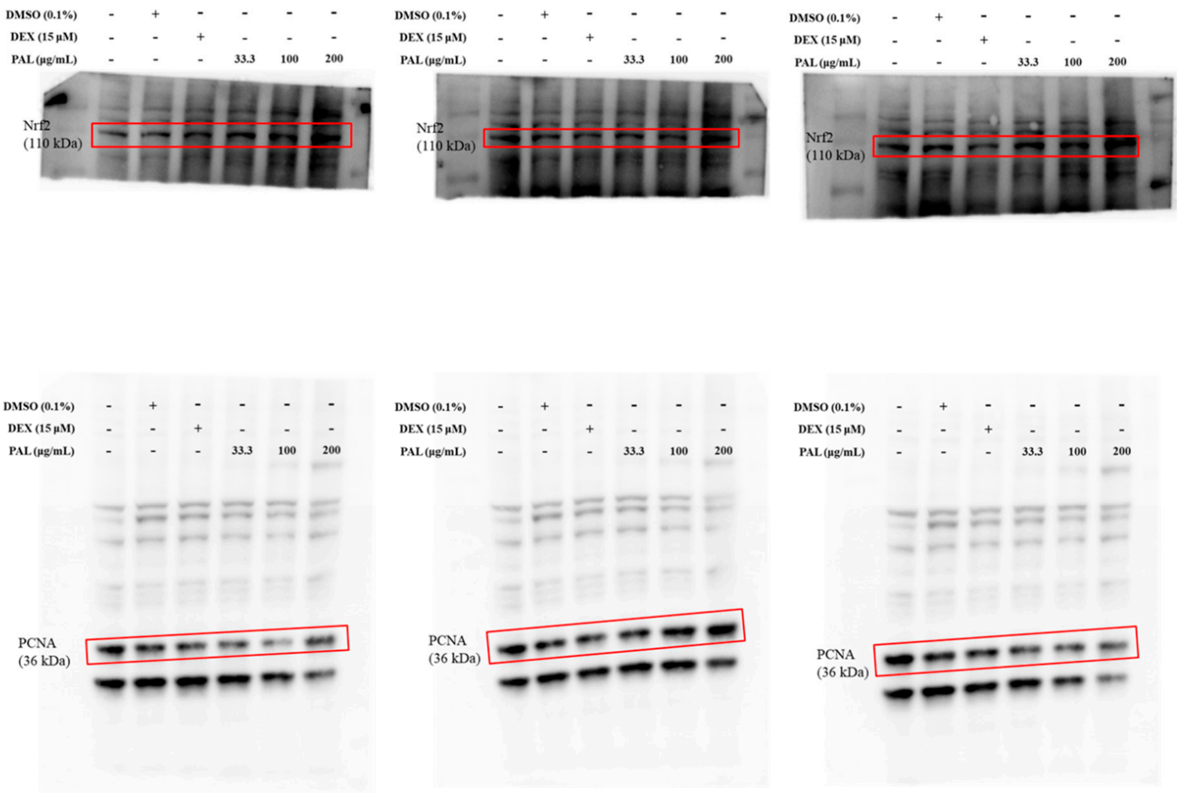
DMSO (0.1%)
DEX (15 µM)
PAL (µg/mL)
33.3
100
200
Nrf2
(110 kDa)
PCNA
(36 kDa)

(E)

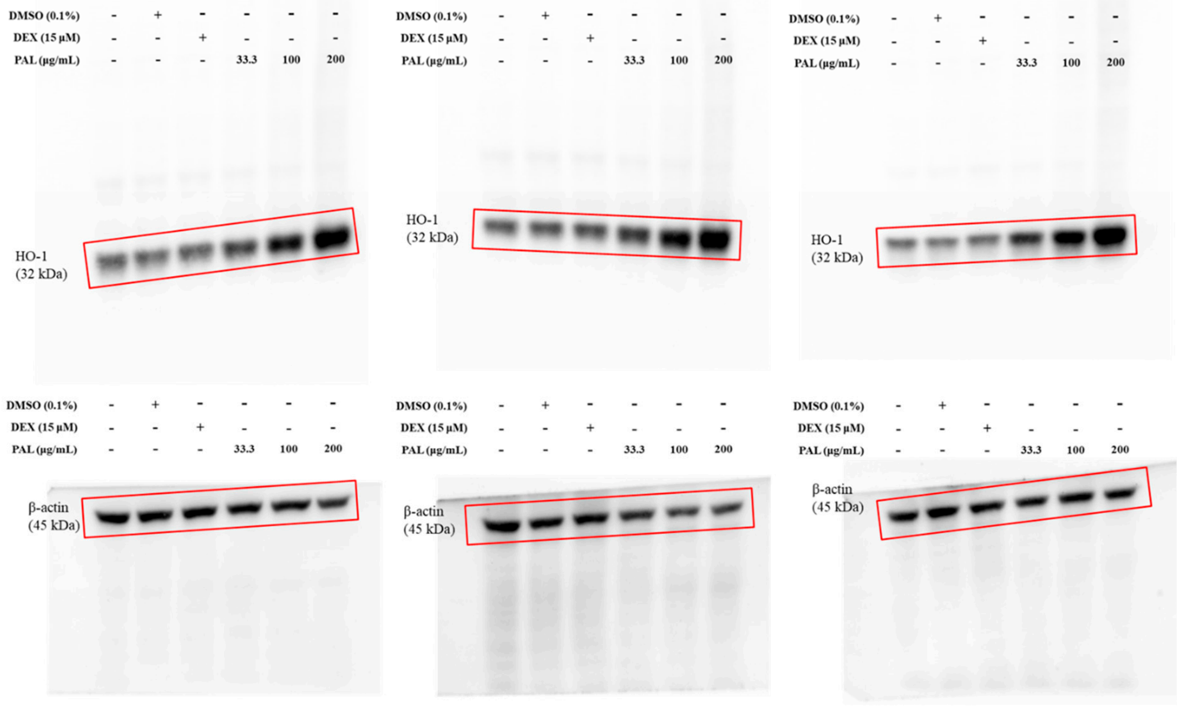
DMSO (0.1%)
DEX (15 µM)
PAL (µg/mL)
33.3
100
200
HO-1
(32 kDa)
β-actin
(45 kDa)

(F)

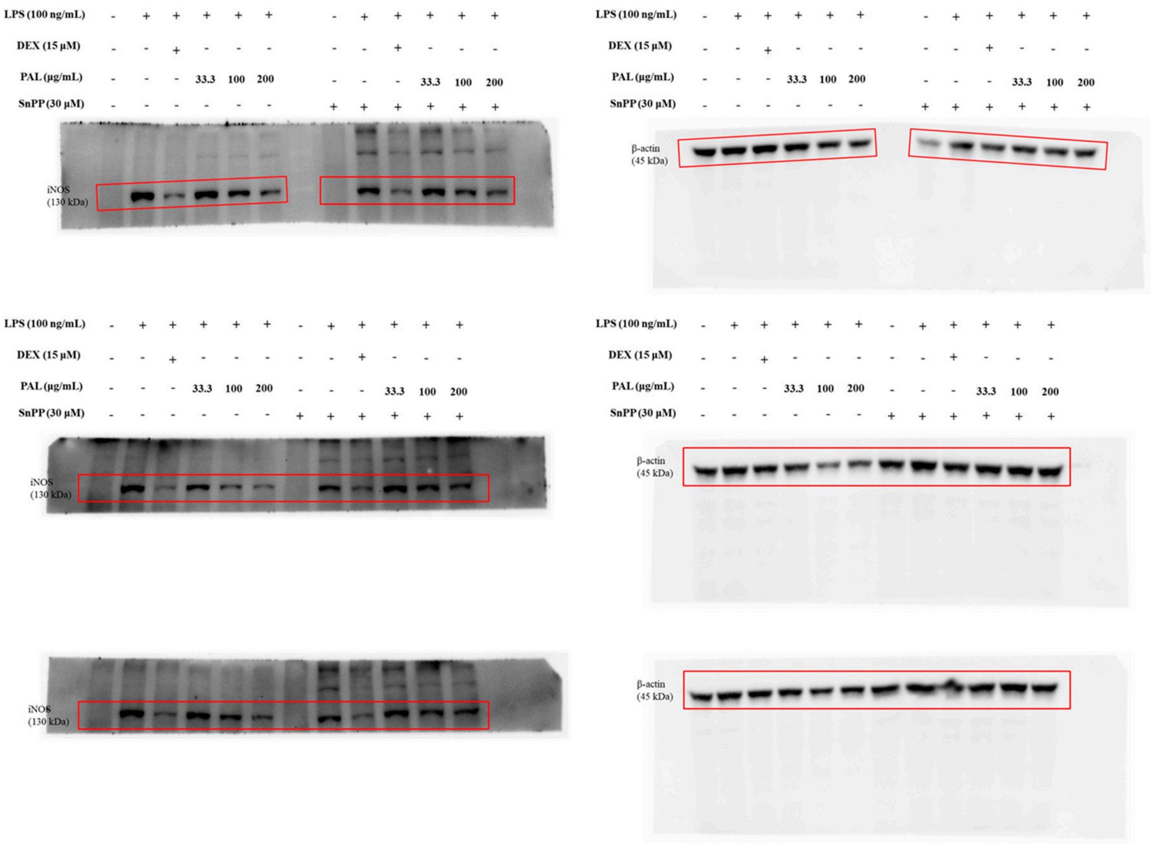

(G)

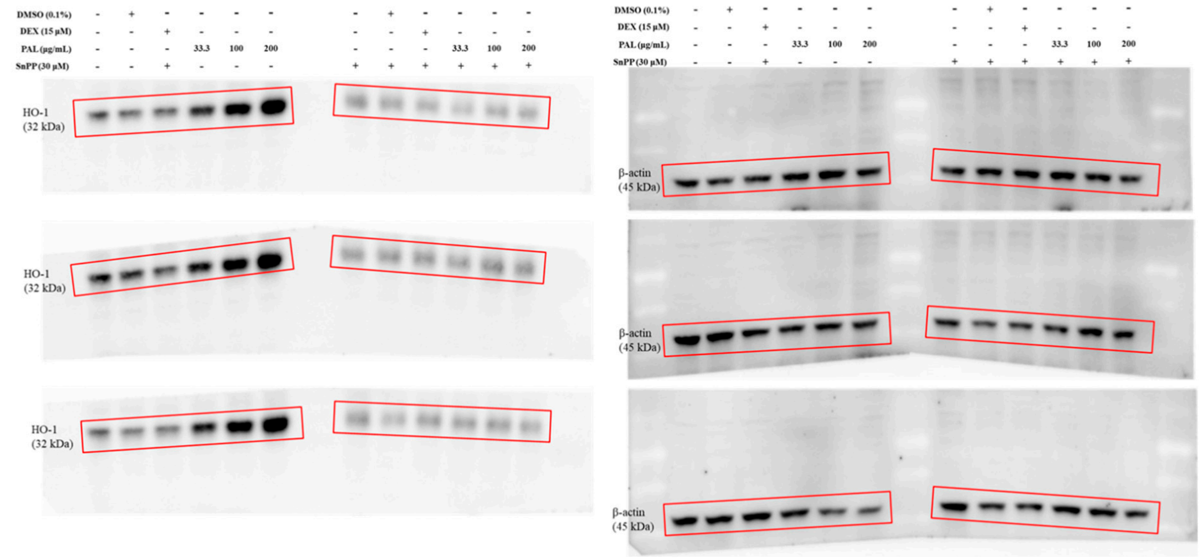

Figure S4. The original Western blotting images. The original blots marked by a red box in main images were used for image acquisition and densitometric analysis. (A) The Western blotting images corresponding to Figure 1D. (B) The Western blotting images corresponding to Figure 2A. (C) The Western blotting images corresponding to Figure 2B. (D) The Western blotting images corresponding to Figure 4A. (E) The Western blotting images corresponding to Figure 4B. (F) The Western blotting images corresponding to Figure 5A. (G) The Western blotting images corresponding to Figure 5C.
